# Chloral in Cancer

**Published:** 1874-11

**Authors:** 


External Application of Chloral in the Treatment of Cancer. M. Constantin Paul employs, with great advantage, suppositories of hydrate of chloral, introduced into the vagina, in the treatment of cancer of the uterus; and in a number of cases in which he has employed this method of treatment in the Pitie and Hopital Saint Antoine, he has always obtained the three following results: Marked diminution of the pain; a favorable change in the cancerous sore; and disappearance of the fœtid odor. The latter fact confirms the anti-putrid and anti-fermentable properties which have recently been attributed to solutions of chloral by MM. Dujardin-Beaumets and Hirne. M. Constantin Paul employs suppositories containing one gramme of chloral enveloped in cacao butter. M. Martineau has also obtained excellent results from the use of chloral in cancer or cancerous sores, either of the breast or uterus. A diminution of the pains, complete disinfection, and, in fine, a very rapid change in the altered surfaces, is obtained. He employs charpie, soaked in a solution of one gramme of chloral dissolved in twenty grammes of water, with which the charpie must be kept constantly moist. M. Martineau has also observed a decrease in the metrorrhagia by the employment of chloral in this way in uterine cancer.—*Bull. Gen. de Ther.*

